# Heritability of carotid intima-media thickness and inflammatory factors of atherosclerosis in a Chinese population

**DOI:** 10.1038/s41598-024-71454-8

**Published:** 2024-09-03

**Authors:** Tsai-Chung Li, Cheng-Chieh Lin, Chiu-Shong Liu, Chih-Hsueh Lin, Shing-Yu Yang, Chia-Ing Li

**Affiliations:** 1 Department of Public Health, College of Public Health, China Medical University, Taichung, Taiwan; 2 Department of Audiology and Speech-Language Pathology, Asia University, Taichung, Taiwan; 3 School of Medicine, College of Medicine, China Medical University, Taichung, Taiwan; 4 Department of Family Medicine, China Medical University Hospital, Taichung, Taiwan; 5 Department of Medical Research, China Medical University Hospital, No. 2, Yude Rd., North Dist., Taichung, 404 Taiwan

**Keywords:** Intima-media thickness, CRP, Heritability, Cardiovascular diseases, Epidemiology

## Abstract

Carotid intima-media thickness (cIMT), a marker of subclinical atherosclerosis, has been found to be associated with incident stroke. High-sensitivity C-reactive protein (CRP) and fibrinogen have been demonstrated to be associated with atherosclerosis. Previous studies on heritability estimates of IMT, CRP, and fibrinogen among Chinese populations are limited. This study aims to estimate the heritability of these risk factors in residents who participated in the Taichung Community Health Study (TCHS) and their family members. A total of 2671 study subjects from 805 families were enrolled in the study, selected from a random sample of TCHS participants and their family members. CRP, and fibrinogen were obtained from each participant, and a questionnaire interview was conducted. cIMT was measured by high-resolution B-mode ultrasound and expressed as the mean of the maximum. Heritability estimates and the familial correlation of cIMT, CRP, and fibrinogen among family pairs were determined with SAGE software. With multivariate adjustments, significant heritability was found for cIMT (h^2^ = 0.26, *P* < 0.001), CRP (h^2^ = 0.34, *P* < 0.001), and fibrinogen (h^2^ = 0.48, *P* < 0.001). The intrafamilial correlation coefficients for the three indexes in the parent–offspring pairs were significant (*P* < 0.001) and ranged from 0.17 to 0.41. The full sibship correlations were also significant (*P* < 0.001) for the three indexes and ranged from 0.19 to 0.47. This study indicates that a moderate proportion of the variability in CRP, fibrinogen, and cIMT can be attributed to genetic factors in Chinese populations. The findings suggest that CRP is associated with cIMT, whereas no significant association exists between fibrinogen and cIMT.

## Introduction

Cardiovascular diseases (CVDs) are the leading cause of global mortality and responsible for about 17.9 million deaths annually^1^. These diseases account for 32% of all deaths worldwide, with 85% of CVD-related deaths attributed to heart attacks and strokes in 2019. The World Health Organization’s 2019 statistics reveal that over three quarters of CVD deaths occur in low- and middle-income countries. Although the age-standardized mortality rates for CVDs have declined in the past two decades^2^, the overall number of individuals affected by and dying from these diseases continues to increase^3,4^. CVD remains to be the leading cause of death in industrialized nations^5^. For instance, ischemic heart disease and stroke were the top two causes of death in the United States and the United Kingdom in 2021^6,7^. Early detection of CVDs is crucial because it enables prompt intervention through counseling and medication management.

Atherosclerosis is a progressive disease that involves the accumulation of lipids and fibrous elements in the arteries, leading to the development of plaque and inflammation. This development ultimately results in the narrowing of the blood vessels, which is known as stenosis. Carotid atherosclerosis specifically refers to the presence of atherosclerotic disease in the carotid arteries. Previous studies have established a link between measurable traits, such as carotid intima-media thickness (cIMT), and increased risk of CVDs^8–14^. cIMT, which is measured through B-mode ultrasound, is the distance between the lumen–intima and media–adventitia interfaces of the carotid artery. This noninvasive method has been widely used and proven to be highly reproducible, repeatable, and sensitive in detecting carotid artery pathologies^15,16^.

Inflammation is a known risk factor for the development of atherosclerosis. C-reactive protein (CRP) and fibrinogen serve as biomarkers of inflammation and are associated with an increased risk of atherosclerosis^17–20^. High levels of CRP have been found to be positively associated with the risk of vascular-related diseases and vascular mortality^17^, and elevated levels of fibrinogen have been shown to predict sudden cardiac death in middle-aged men (aged 42–61 years)^18^ and mortality in long-term stroke survivors^19^.

Many studies on estimating the heritability of CRP, fibrinogen, and cIMT have been conducted in the United States, Europe and Latin America^21–78^ (Supplementary Table 1). However, similar studies on the Chinese population are limited; only one study on cIMT has been conducted on a Chinese population^65^. In a study of Chinese adults^65^, a total of 62 families (360 subjects) were included, but the analysis of heritability did not adjust for factors, such as age, gender, and lifestyle behaviors. The current study aims to estimate the heritability of CRP, fibrinogen, and cIMT in family members of Taiwanese individuals residing in a community, and to explore the associations between CRP and fibrinogen with cIMT.

## Methods

### Participants and study design

The Taichung Community Health Study (TCHS) involves a longitudinal population-based cohort. It was initiated in 2004 to prospectively explore the cardiovascular risk factors related to metabolic syndrome in Taiwan. The detailed methodology of TCHS is described elsewhere^79^. In brief, the first wave survey of TCHS consisted of 2359 adults aged 40 years and above who were randomly selected from the general population of Taichung. The second wave survey involved 1666 residents longitudinally selected from 2007 to 2009. In the third wave survey, the TCHS cohort was adopted, and their family members, including spouses, parents, children, and siblings, were invited to participate in the study from 2010 to 2013. The exclusion criteria were participants who did not provide any family members. All participants were followed up again in 2017. A total of 805 families with 2671 subjects were recruited. The average number of persons assessed in each family was 3.3, and the size of the families was 2–14 persons. The study was approved by the Human Research Committee of China Medical University Hospital (DMR98-IRB-323) and all methods were performed in accordance with the relevant guidelines and regulations. Written informed consent was obtained from each participant.

### Measures

#### cIMT

cIMT was measured using noninvasive, high-resolution, B-mode ultrasonography. After resting in the supine position with the neck slightly hyperextended for at least 10 min, all the study subjects underwent carotid ultrasound examination using a 7.5-MHz probe (GE L7000, GE, Milwaukee, Wis., the USA) to scan the near and far walls of arterial segments bilaterally. This procedure allowed longitudinal (anterior oblique, lateral, and posterior oblique) and transverse views to be obtained. Each ultrasound image was recorded on a computer with an online digital filing system, and the thickness of the intima-media complex and the presence of atherosclerotic plaques were measured. cIMT was primarily measured on the far wall of the common carotid artery (CCA) proximal to the CCA bifurcation and the carotid bulb and internal carotid artery. The cIMT measurements were manually outlined over a distance of 1 cm at each respective site. The maximum value of the cIMT measurement at each site was used in this study. The intra-operator reliability of cIMT was 0.85 and 0.97 for the two operators, respectively, while the inter-operator reliability was 0.88.

#### Laboratory examination

Blood was drawn with minimal trauma from the antecubital vein in the morning after a 12-h overnight fast and sent for analysis within 4 h of collection. High-sensitivity C-reactive protein (hs-CRP) and fibrinogen levels were measured using nephelometry, which is a latex particle-enhanced immunoassay (TBA-200FR, Tokyo, Japan). The inter-assay and intra-assay coefficients of variation (CVs) for hs-CRP were < 2.0% and 1.9%, respectively, with a lower detection limit of 0.1 mg/L. The inter-assay and intra-assay CVs for fibrinogen were 3.6% and 6.1%, respectively. The Clinical Laboratory Department of China Medical University Hospital uses a fully automatic biochemical autoanalyzer (Unicel DxC 800 Synchron Clinical System; Beckman Coulter, Fullerton, CA, the USA) to analyze biochemical markers, such as fasting plasma glucose, triglyceride, and low-density lipoprotein-cholesterol (LDL-L).

#### Anthropometric measurement

Weight and height were measured using an automatic anthropometer (Super-view, HW-666). The participants were barefoot and wore light clothing. The body mass index (BMI) was calculated using the formula weight (kg) divided by height squared (m^2^). Waist circumference (WC) was measured with the participant standing by using a tape measure. The measurement was obtained at the midway point between the inferior margin of the last rib and the crest of the ilium on a horizontal plane. WC was measured after the participant exhaled; the tape was placed snugly (but not compressing the skin) just above the hipbones. Blood pressure was measured using an electronic device (COLIN, VP-1000, Japan). The participants were seated and did not encounter any distractions. Two measurements were taken in the right arm of each participant. A properly sized cuff and a standard tunnel-type electronic sphygmomanometer (OMRON, HBP-9020, Japan) were used. The participants were instructed to rest and sit upright on a chair next to a table for 5–10 min. They were advised to keep their arm in a comfortable position at the heart level, with their back against the chair and with their legs uncrossed. The participants were also instructed to rest their forearm on the table, with their palm facing upward.

#### Health-related practices and disease history

The study participants who engaged in regular physical activities were defined as those who participated in leisure-time activities for a minimum of 30 min once a week in the past 6 months. An assessment was performed through a single item regarding leisure-time activity habits. Smoking status was categorized as either former smokers, current smokers, and never smoked. Former smokers were individuals who had smoked at least 100 cigarettes during their lifetime but were not currently smoking at the time of the interview. Alcohol drinking status was classified as former drinkers, current drinkers, or never drank. Former drinkers were individuals who had regularly consumed alcohol for a minimum of 12 months during their lifetime but did not consume alcohol at the time of the interview. Information on the presence of menopause was collected through self-report question. A checklist of chronic conditions was utilized to assess various health conditions, such as hypertension, hyperlipidemia, diabetes, stroke, and heart disease.

### Statistical analysis

Descriptive statistics were utilized to analyze various aspects of the study participants, including their demographic characteristics, menopause, disease history, biomarkers (WC and BMI), hs-CRP levels, fibrinogen levels, and cIMT. Given the right-skewness observed in the distributions of the hs-CRP, fibrinogen, and cIMT levels, natural log-transformation was employed to achieve data normalization. Participants from TCSH were considered probands. Family relationships were categorized into five types: parent, sibling, offspring, spouse, and grandchildren.

The Statistical Analysis for Genetic Epidemiology program (S.A.G.E. [2021] Statistical Analysis for Genetic Epidemiology, Release 6.4.2: http://darwin.cwru.edu) was used to calculate familial correlations and the heritability of log-transformed hs-CRP, fibrinogen, and cIMT. The intraclass correlations of spouses, siblings, parents, offspring, and grandchildren and their standard errors were calculated by the FCOR procedure in S.A.G.E. A variance component method that partitions the observed covariance among relatives into genetic and environmental components was used to estimate the heritability of log-transformed hs-CRP, fibrinogen, and cIMT by using the ASSOC procedure in S.A.G.E. In a variance component framework, the variance in a quantitative trait is assumed to be due to additive genetic (σ^2^_g_) and random environmental (σ^2^_e_) effects, and the heritability of the trait is obtained by the ratio σ^2^_g_ /(σ^2^_g_ + σ^2^_e_). Linear mixed models were used to assess the associations among hs-CRP, fibrinogen, and cIMT and the random effect was used to consider the dependence of family members. The analyses were performed using SAS version 9.4 (SAS Institute Inc., Cary, NC) with a significance level of 0.05 (two-sided).

## Results

A total of 2671 study subjects with complete data were included. The 2671 subjects belonged to 805 families with an average family size of 3.3 (standard deviation, 1.4). The relationships consisted of spouse (370 couples), sibships (982 full sibling pairs), 1823 parent–offspring pairs, and 191 grandparent–grandchild pairs.

Table 1 shows the demographic, medical history, anthropometric characteristics, and laboratory characteristics of the participants. The estimates of the heritability of hs-CRP, fibrinogen, and cIMT with and without the covariates are presented in Table 2. The highest heritability estimate without the covariates was for fibrinogen (h^2^ = 0.45, *P* < 0.001), followed by hs-CRP (0.29, *P* < 0.001), and the lowest was for cIMT (0.07, *P* < 0.01). When age and gender were considered, the corresponding heritability estimates for fibrinogen, hs-CRP, and cIMT were 0.49 (*P* < 0.001), 0.30 (*P* < 0.001), and 0.26 (*P* < 0.001), respectively. When health behavior, disease history, and biomarkers were considered further, the corresponding heritability estimates for fibrinogen, hs-CRP, and cIMT were similar (0.48, 0.34, and 0.26, respectively; all *P* < 0.001).

**Table 1:** Characteristics of spouse, parents, offspring, grandparent, grandchild and sibling of probands.

Measurements	Spouse (n = 740)	Parents (n = 1156)	Offspring (n = 1236)	Grandparent (n = 109)	Grandchild (n = 147)	Sibling (n = 1207)
Age (years)^a^	60.8 (10.4)	62.2 (11.3)	34.8 (12.8)	76.5 (6.2)	20.4 (5.9)	42.6 (15.9)
Female	50%	56%	50%	56%	44%	58%
Menopause	38%	44%	9%	56%	0.7%	24%
Disease history						
Heart disease	14%	16%	3%	28%	1%	5%
Hypertension	33%	35%	8%	57%	1%	14%
Diabetes mellitus	13%	14%	3%	19%	0%	5%
Hypercholesterolemia	24%	25%	12%	22%	5%	15%
Stroke	3%	3%	0.2%	6%	0%	1%
Health behaviors						
Current smoking	11%	10%	14%	6%	7%	12%
Drinking	18%	16%	14%	11%	5%	14%
Regular exercise	71%	70%	50%	74%	63%	57%
Anthropometric characteristics						
Waist circumference (cm)^a^	83.5 (9.6)	83.4 (9.8)	79 (11.4)	85 (10.3)	76.2 (12.5)	79 (10.5)
Body mass index (kg/m^2^)^a^	24.4 (3.1)	24.4 (3.5)	23.3 (4.1)	24.3 (3.5)	22.2 (4.7)	23.2 (3.8)
Systolic blood pressure (mmHg)^a^	126.8 (16.1)	128.1 (16.7)	115.2 (14.4)	136.3 (18.3)	112.3 (12.2)	117.8 (15.6)
Diastolic blood pressure (mmHg)^a^	77.7 (9.9)	77.8 (10.1)	74.0 (10.8)	75.3 (10.4)	69.5 (8.8)	74.7 (10.7)
LDL cholesterol (mg/dl)^a^	119 (30.8)	118.6 (30.9)	110.2 (31.6)	110.2 (32.2)	98.5 (31.4)	114.2 (32.1)
Triglycerides (mg/dl)^b^	100.5 (34.2, 295.3)	102.5 (34.9, 301.3)	84.8 (26.2, 274.8)	98.5 (37.0, 262.4)	70.8 (25.1, 200.1)	89.1 (28.0, 283.3)
Fasting glucose (mg/dl)^a^	105.3 (25.9)	105.3 (26.6)	94.1 (14.0)	108.3 (35.1)	90.5 (7.6)	97 (20.0)
Biomarkers						
Hs-CRP (mg/dl)^b^	0.1 (0.0, 0.8)	0.1 (0.0, 0.8)	0.1 (0.0, 0.6)	0.1 (0.0, 1.4)	0 (0.0, 0.3)	0.1 (0.0, 0.6)
cIMT (mm)^b^	1.5 (0.6, 3.6)	1.5 (0.6, 3.7)	0.8 (0.4, 1.8)	2.1 (0.9, 4.8)	0.7 (0.4, 1.0)	1.0 (0.4, 2.4)
Fibrinogen (mg/dl)^b^	357.8 (246.6, 519.3)	365.0 (251.5, 529.7)	340.4 (234.5, 493.9)	372.4 (261.7, 530.0)	327.0 (229.8, 465.4)	347.2 (239.3, 503.9)

LDL cholesterol, Low density lipoprotein-cholesterol; Hs-CRP: High sensitivity C-reactive protein; cIMT: Carotid intima-media thickness.

^a^Data were shown as mean (standard deviation).

^b^Data were shown as geometric mean (95% of confidence interval of geometric mean) due to skew distribution.

**Table 2:** Familial correlations and heritability estimation of hs-CRP, fibrinogen, and cIMT.

	Log-transformation of hs-CRP		Log-transformation of fibrinogen		Log-transformation of cIMT	
	N pairs	Estimate (95% CI)	N pairs	Estimate (95% CI)	N pairs	Estimate (95% CI)
Familial correlations						
Spouse	370	0.09 (− 0.01, 0.19)	369	0.20 (0.10, 0.30)***	358	0.31 (0.21, 0.40)***
Sibling	982	0.19 (0.11, 0.27)***	982	0.30 (0.22, 0.38)***	896	0.47 (0.39, 0.54)***
Parent-offspring	1823	0.17 (0.11, 0.23)***	1819	0.28 (0.22, 0.34)***	1666	0.41 (0.35, 0.47)***
Grandparent	191	0.16 (0.02, 0.29)*	191	0.03 (− 0.13, 0.18)	170	0.20 (− 0.04, 0.42)
Heritability						
No covariate		0.29 (0.21, 0.37)***		0.45 (0.38, 0.53)***		0.07 (0.00, 0.15)*
Age and sex adjusted		0.30 (0.22, 0.38)***		0.49 (0.42, 0.56)***		0.26 (0.18, 0.35)***
Age, sex, menopause, health behavior, disease history and biomarkers adjusted^a^		0.34 (0.26, 0.42)***		0.48 (0.41, 0.56)***		0.26 (0.17, 0.35)***

Hs-CRP: High sensitivity C-reactive protein; cIMT: Carotid intima-media thickness; CI, Confidence interval.

^a^Health behavior included smoking, drinking, and regular exercise. Disease history included heart disease, hypertension, diabetes mellitus, hypercholesterolemia, and stroke. Biomarkers included waist circumference and body mass index.

*P < 0.05; ***P < 0.001.

The familial correlation coefficients for spouse, sibling, parent–offspring, and grandparent–grandchildren relationships for hs-CRP, fibrinogen, and cIMT are shown in Table 2. For hs-CRP, the lowest familial correlation coefficients were observed for spouse (*r* = 0.09), and significant weak familial correlation coefficients were found for sibling, parent–offspring, and grandparent–grandchildren relationships (0.16–0.19). For fibrinogen and cIMT, the lowest familial correlation coefficients were observed for grandparent–grandchildren relationships (0.03 for fibrinogen and 0.20 for cIMT), and significant weak-to-moderate familial correlation coefficients were found for the other types of family relationships (0.28–0.30 for fibrinogen and 0.41–0.47 for cIMT).

**Fig. 1:**
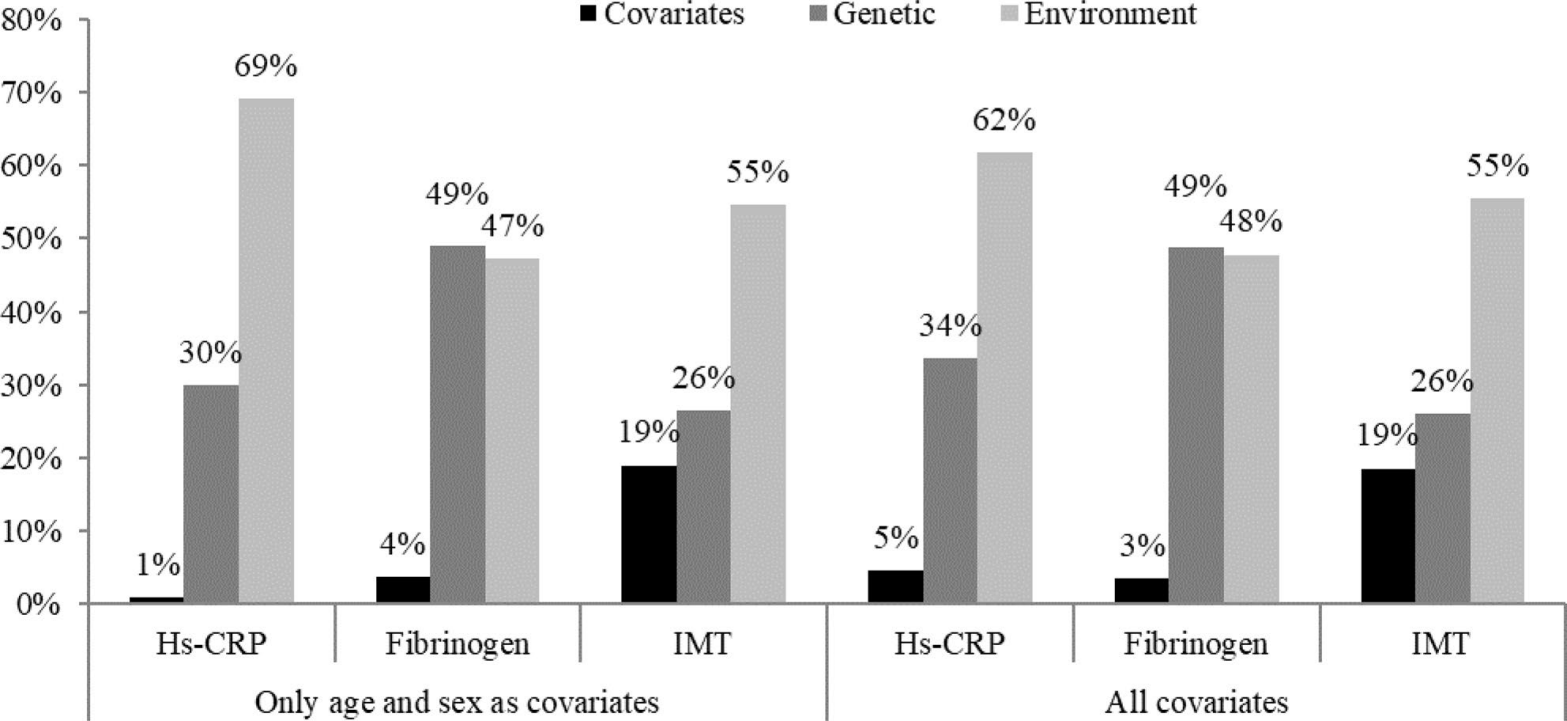
Percentage of total variance explained by covariates, genetic factors, and random environment. All covariates included health behavior (smoking, drinking, regular exercise), disease history (heart disease, hypertension, diabetes mellitus, hypercholesterolemia, stroke), and biomarkers (waist circumference, body mass index).

Figure 1 illustrates the proportion of variance accounted for by the covariates, genetic factors, and environmental factors. When age and gender were considered, fibrinogen exhibited the highest percentage of variance attributed to genetic factors (49%), followed by hs-CRP (30%) and cIMT (26%). After lifestyle behavior, disease history, and biomarkers were incorporated into the analysis, the percentages of the variance attributed to the genetic factors for fibrinogen and cIMT did not change (49% and 26%, respectively), whereas the percentage of the variance attributed to the genetic factors for hs-CRP increased to 34%. The covariates explained the majority of the variance for cIMT (19%), followed by hs-CRP (5%) and fibrinogen (3%).

Table 3 presents the associations among hs-CRP, fibrinogen, and cIMT after considering gender, age, menopause, disease history, smoking status, alcohol drinking status, physical activity, anthropometric characteristics, and biomarkers. A significant positive association was observed between hs-CRP and fibrinogen. The log-fibrinogen was a significant factor of log-hs-CRP (β = 2.981, *P* < 0.001), while log-hs-CRP was a significant factor of log-fibrinogen (β = 0.091, *P* < 0.001). However, only log-hs-CRP, and not log-fibrinogen, was significantly associated with log-cIMT (β = 0.018, *P* = 0.003).

**Table 3:** The associations among hs-CRP, fibrinogen, and cIMT considering the dependence of family members in generalized linear mixed models.

	Model 1		Model 2		Model 3	
	Log-transformation of hs-CRP		Log-transformation of fibrinogen		Log-transformation of cIMT	
	β ± SE	*P* value	β ± SE	*P* value	β ± SE	*P* value
Log-hs-CRP	–	–	0.091 ± 0.002	< 0.001	0.018 ± 0.006	0.003
Log-fibrinogen	2.981 ± 0.071	< 0.001	–	–	0.007 ± 0.033	0.843

β ± SE indicates regression coefficient and its standard error. All models were adjusted for kinship, gender, age, menopause, heart disease, hypertension, diabetes, hyperlipidemia, stroke, smoking status, alcohol drinking, exercise, waist circumference, and body mass index. The log-transformed response variables were analyzed in the table.

Hs-CRP: High sensitivity C-reactive protein; cIMT: Carotid intima-media thickness.

## Discussion

In this study involving community-based participants and their family members, the inflammation markers, such as CRP, fibrinogen, and cIMT, exhibited heritability. Among these markers, fibrinogen displayed the highest heritability estimate, followed by CRP and cIMT. The results indicated a significant and moderately sized heritability of CRP, fibrinogen, and cIMT. Although these factors may have been influenced by various environmental and genetic factors, the evidence suggests a substantial genetic contribution to the variation observed in these markers (26% for cIMT, 34% for CRP, and 49% for fibrinogen) even after accounting for the effects of age, gender, and lifestyle factors. Our study also identified factors that were significantly associated with CRP, fibrinogen, and cIMT in Chinese individuals residing in the community.

Although several studies have reported the familial heritability of CRP, fibrinogen, and cIMT across various populations^21–78^, including Japanese Americans for CRP^23^, the Koreans for cIMT^76^, and Vietnamese for both CRP and fibrinogen^41^, only a limited number of studies have specifically focused on the Chinese population for cIMT^65^. Supplementary Table 1 summarizes the methods and outcomes of prior pedigree and twin studies. Chien et al.^65^ estimated the heritability and familial correlation of spouse, parent–offspring, and sibling with carotid atherosclerosis in 62 families of 360 ethnic Chinese subjects on the basis of adolescent primary hypercholesterolemia probands^12^. Their results revealed that the estimated heritability of IMT is 59% after age, HDL, and systolic blood pressure are considered. The estimated heritability in the current study is 26%, which is lower than that in Chien et al.’s study^65^. The difference between the current study and Chien et al.’s work may be due to two reasons. The first one is related to the covariates considered in the estimation. The covariates considered in Chien et al.’s study were age, high-density lipoprotein cholesterol, and systolic blood pressure; those considered in the present study were age, gender, smoking status, drinking status, regular exercise, heart disease, hypertension, diabetes, hypercholesterolemia, stroke, WC, and BMI. The second reason is that in Chien et al.’s study, the subjects had primary hypercholesterolemia, whereas in the current study, the subjects were participants residing in a community. Some studies that used family study designs to estimate IMT for other populations obtained values ranging from 16 to 92%^38,53–73^. These variations in heritability estimates for IMT may be due to various factors, such as ethnic populations, populations with specific chronic conditions, and the inclusion of different covariates in the estimation process. With regard to the familial correlation among different family relationships, our findings are consistent with those reported by Chien et al.^65^. In Chien et al.’s study, the correlation coefficients for spouse, parent–offspring, and sibling were 0.39, 0.38, and 0.35, respectively; in the current study, the corresponding correlation coefficients were 0.31, 0.41, and 0.47, respectively.

With regard to heritability estimates for CRP, previous studies that used family study designs were conducted on various populations, including Japanese Americans^23^, Caucasian and African Americans with type 2 diabetes^29^, four US communities^21^, and African Americans^31^. The estimated heritability for CRP in these studies ranged from 10 to 45%. The current study obtained a CRP heritability estimate of 34%, which is consistent with the result of the study conducted on Japanese Americans where the heritability was reported to be 30%^23^. Notably, the study on Japanese Americans reported a familial correlation of 0.2 for first-degree relatives, which is similar to the familial correlations observed in the current study (0.17 for parent–offspring and 0.19 for siblings).

Previous studies that estimated the heritability of fibrinogen in Asia population are relatively few^24,26,28,33,34,43,45–50^. One study that used a quantile-specific approach on participants from the Framingham Heart Study reported fibrinogen heritability values ranging from 0.30 to 0.65 for the offspring–parent relationship and from 0.28 to 0.50 for full siblings across different percentiles^50^. Another study conducted in Sweden found a fibrinogen heritability of 51% in 85 families identified from probands with early myocardial infarction and 85 families from the general population^43^. Yet another two studies employed a twin study design based on the Danish Twin Registry and reported a fibrinogen heritability estimates of 21% in 282 Danish twins^36^ and 34% in 285 Danish twins^52^. The current study’s findings are more consistent with those of the family-based studies^33,43,50^ than with those of the twin study^36,42,52^.

Previous population-based studies presented conflicting findings regarding the relationship of CRP and fibrinogen with the atherosclerosis index of cIMT^80–82^. For instance, a large cohort study with 2502 subjects reported that the combination of CRP and fibrinogen levels is associated with multivariate adjusted cIMT^80^. However, this study focused on exploring the joint effects of CRP, fibrinogen, and smoking and did not differentiate the independent effects of CRP and fibrinogen. In another study specifically conducted on men, CRP and fibrinogen did not exhibit a significant association with cIMT after adjusting for BMI^81^. Similarly, a study that adopted a community-based sample without advanced atherosclerotic disease demonstrated the independent effect of CRP on cIMT but found that this effect disappears after controlling for fibrinogen levels^82^. In the current family study, a link was observed between CRP and fibrinogen. Furthermore, when CRP and fibrinogen were considered simultaneously, only CRP remained significantly associated with cIMT. Two reasons could explain why previous studies failed to establish a significant association between CRP and cIMT. First, previous studies used CRP instead of CRP, which is known to be less sensitive than CRP. Second, previous studies had smaller sample sizes compared with the sample size in the current study (*n* = 900 in the study of Nagasawa et al.^81^ and *n* = 1018 in the study of Sitzer et al.^82^).

We observed that when covariates other than age and sex were added, the contribution of environmental factors either slightly changed or remained unchanged. Specifically, the proportions of total variance in CRP, fibrinogen, and cIMT explained by environmental factors shifted from 69 to 62%, 47 to 48%, and 55 to 55%, respectively. This suggests that the influence of additional covariates such as lifestyle behaviors, disease history, and biomarkers on the variability of CRP, fibrinogen, and cIMT is accounted for by age and sex.

The present study has several limitations that need to be acknowledged. First, this study’s subjects primarily consisted of Han Chinese individuals, so the generalizability of the findings to other racial and ethnic groups may be limited. The extent to which these heritability estimates can be applied to other populations depends on the association between IMT and genetic variants and the variation in the prevalence of these variants across different ethnic groups. Second, the current analysis did not include data on passive smoking status and dietary factors, which are important determinants of IMT. These data were excluded because of their unavailability. However, the Taiwanese government implemented a smoking prohibition policy in public settings in 2009, which led to a decrease in the prevalence of passive smoking from 29.3 to 14.6% in family settings and from 33.2 to 18.2% in occupational settings between 2005 and 2010^83^. Therefore, the confounding effect of passive smoking on our heritability estimates is likely to be small, given its low prevalence. In terms of dietary factors, research has indicated that a gourd/root vegetable diet is associated with an increase in cIMT in the Bangladeshi population, whereas a balanced diet is associated with a decrease in IMT^84^. Furthermore, a randomized controlled trial demonstrated that improving dietary quality by increasing fruit, vegetable, and dairy intake in individuals with well-controlled type 1 and type 2 diabetes can slow the progression of common carotid artery IMT^85^. Therefore, the lack of consideration of dietary factors in the present study may affect the estimates of heritability. Third, the current analysis assumed the absence of gene–covariate interactions during the estimation of the heritability of biomarkers, that is, heritability was assumed to be constant across different subgroups defined by covariates, such as age and gender. Although this assumption was commonly used extant studies, it may be violated if gene–covariate interactions are present. Fourth, epigenetic factors and shared environments may contribute to heritability estimates, but this study did not consider shared environments. Failure to consider shared environments could lead to an overestimation of the genetic contribution in heritability estimates. However, about 90% of the siblings in this study indicated that they lived in different households and were separated either from each other or from their parents. Early shared environments could have contributed to the correlations observed for cIMT, CRP, and fibrinogen among adult relatives. With regard to spouses, determining how much of their correlation is due to shared environments versus assortative mating for factors related to cIMT, CRP, and fibrinogen is difficult. The correlation coefficients for cIMT, CRP, and fibrinogen were weaker between spouses than between siblings and parent–offspring but stronger than the correlation between grandparents. According to the findings, genetic factors largely explained the variation of these factors, especially CRP and fibrinogen, given the high heritability estimates.

## Conclusion

This study indicates that a moderate proportion of the variability in CRP, fibrinogen, and cIMT can be attributed to genetic factors in Chinese populations. Specifically, in the different family relationships, cIMT showed a high familial correlation, whereas CRP and fibrinogen had low correlations. These risk factors were influenced by environmental factors, such as smoking, alcohol consumption, physical activity, and obesity. The findings suggest that CRP is associated with cIMT, whereas no statistically significant association exists between fibrinogen and cIMT.

## Supplementary Information

Supplementary Table S1.

## Data Availability

The datasets generated during and/or analyzed during the current study are available from the corresponding author on reasonable request.

## Abbreviations

CVDs: Cardiovascular diseases
cIMT: Carotid intima-media thickness
CRP: C-reactive protein
TCHS: Taichung Community Health Study
CCA: Common carotid artery
hs-CRP: High-sensitivity C-reactive protein
CVs: Coefficients of variation
LDL-C: Low-density lipoprotein-cholesterol
BMI: Body mass index
WC: Waist circumference
SAGE: Statistical Analysis for Genetic Epidemiology
