# Supplementary material for: Heritability of carotid intima-media thickness and inflammatory factors of atherosclerosis in a Chinese population
Source: Sci Rep. 2024 Sep 3;14:20440. doi: 10.1038/s41598-024-71454-8 (PMC11371917; doi:10.1038/s41598-024-71454-8)
Supplement: Supplementary file 1 — Supplementary Table S1. [file 41598_2024_71454_MOESM1_ESM.docx]

Supplementary Table 1. Intra-familial correlation coefficient and heritability estimates of CRP, fibrinogen, and cIMT from family and twin studies

| Outcome | Author/year | Study country or Study population  (Type of design) | Subjects | Intra-familial correlation coefficient | Heritability | Adjustment for covariates in heritability estimation1 |
| --- | --- | --- | --- | --- | --- | --- |
| CRP | [[21](#_ENREF_21)] Pankow et al., 2001 | USA  (Family study) | 1,253 families from 4 US communities, comprising 2,353 individuals | \| Type \| Pairs \| Coefficient ±SE \| \| --- \| --- \| --- \| \| Spouse \| 120 \| 0.02±0.09 \| \| Pa-off \| 929 \| 0.15±0.04 \| \| Sibling \| 2654 \| 0.20±0.03 \| | 40% | Age and sex |
| CRP | [[22](#_ENREF_22)] Vickers et al., 2002 | England  (Family study) | 98 Caucasian families with essential hypertensive probands comprising 588 individuals | — | 39% | Age and BMI |
| CRP | [[23](#_ENREF_23)] Austin et al., 2004 | Japanese Americans  (Family study) | 68 extended kindreds of Japanese Americans comprising 562 individuals | \| Type \| Pairs \| Coefficient (95% CI) \| \| --- \| --- \| --- \| \| Spouse \| 45 \| 0.18 (-0.15, 0.51) \| \| Pa-off \| 300 \| 0.21 (0.05, 0.37) \| \| Sibling \| 396 \| 0.20 (0.02, 0.38) \| | 30% | Age, sex, BMI, cigarette smoking status, high blood pressure, diabetes, myocardial infarction or coronary artery disease, CRP lowering medications, oral contraceptive and hormone replacement therapy in women |
| CRP | [[24](#_ENREF_24)] Best et al., 2005 | American Indians  (Family study) | 1,294 American Indian relative pairs comprising 1,393 individuals | — | 46% | Age, sex, cohort center, history of cardiovascular disease, physical activity, percent body fat, waist-hip ratio, blood pressure, smoking, diabetes status, impaired glucose, triglyceride, LDL-C |
| CRP | [[25](#_ENREF_25)] Dupuis et al., 2005 | USA  (Family study) | 304 extended families comprising 1,054 individuals | — | 28% | Age, sex, BMI, smoking, cardiovascular disease, fasting glucose level, systolic blood pressure, ratio of total to high-density lipoprotein cholesterol levels, hypertension treatment, lipid reduction treatment and hormone replacement therapy |
| CRP | [[26](#_ENREF_26)] Friedlander et al., 2006 | Israel  (Family study) | 142 kindreds comprising 881 individuals | — | 3% (polygenic effect) | Age and sex |
| CRP | [[27](#_ENREF_27)] Tang et al., 2006 | USA  (Family study) | 349 families comprising 702 nondiabetic white individuals | — | 43% | Age, sex, field center, hormone replacement therapy, smoking, drinking, energy intake, dietary fat, physical activity, and sedentary behavior |
| CRP | [[28](#_ENREF_28)] Saunders et al., 2006 | England  (Family study) | 9,237 families from a national health survey, comprising 22,297 individuals | \| Type \| Pairs \| Coefficient ±SE \| \| --- \| --- \| --- \| \| Spouse \| 1,242 \| 0.08±0.03 \| \| Pa-off \| 896 \| 0.16±0.03 \| \| Sibling \| 117 \| 0.08±0.09 \| | 10% | — |
| CRP | [[29](#_ENREF_29)] Lange et al., 2006 | Caucasian Americans and African Americans (Family study) | 278 Caucasian American and African American families with type 2 diabetes comprise a total of 701 individuals | — | 36% | Age, sex, race, diabetes, drug use, BMI, and smoking |
| CRP | [[30](#_ENREF_30)] Berrahmoune et al., 2007 | French  (Family study) | 320 nuclear France families comprising 1,223 individuals | \| Intra-familial correlation coefficient   \| Type \| Pairs \| Coefficient ±SE \| \| --- \| --- \| --- \| \| Spouse \| 1,242 \| 0.149±0.054 \| \| Pa-off \| 896 \| 0.114±0.033 \| \| Sibling \| 117 \| 0.196±0.060 \| \| \| --- \| --- \| --- \| --- \| --- \| --- \| --- \| --- \| --- \| --- \| --- \| --- \| --- \| | 0.0% (polygenic effect) | Age, sex, BMI, alcohol, smoking, glucose, triglyceride, contraceptive and anti-inflammatory drug |
| CRP | [[31](#_ENREF_31)] Fox et al., 2008 | African Americans (Family study) | 246 families of African Americans comprising 1,317 individuals | — | 45% | Age, sex, and BMI |
| CRP | [[32](#_ENREF_32)] Wu et al., 2009 | African Americans (Family study) | 1,741 white sibling pairs and 355 African American sibling pairs | — | White 31%;  African American 53% | — |
| CRP | [[33](#_ENREF_33)] Neijts et al., 2013 | Netherland (Family study) | 3,534 twins, 1,568 of their non-twin siblings, and 2,227 parents from 3,095 families | — | 45% | Age, sex, BMI, smoking status, current and recent illness, anti-inflammatory medication, female sex hormone status, month of data collection, and batch processing |
| CRP | [[34](#_ENREF_34)] Reding-Bernal et al., 2017 | Mexico  (Family study) | 32 extended families comprising 585 individuals | — | 38% | Age, sex and BMI |
| CRP | [[35](#_ENREF_35)] Retterstol et al., 2003 | Norway  (Twin study) | 155 MZ twins (68 male pairs and 87 female pairs) | Within-pair correlation coefficient: 0.4 | — |  |
| CRP | [[36](#_ENREF_36)] de Maat et al., 2004 | Denmark  (Twin study) | 282 Danish twins (129 MZ pairs, 153 dizygotic same-sex twins’ pairs) | Intra-correlation coefficient ICC (95% CI)  MZ pairs, 0.30 (0.25, 0.30)  DZ pairs, 0.20 (0.19, 0.21) | 22% | Age, sex and BMI |
| CRP | [[37](#_ENREF_37)] MacGregor et al., 2004 | England  (Twin study) | 310 female twin pairs (146 MZ twins and 164 DZ twins) | — | 52% | Age, BMI, systolic blood pressure, and the use of hormone replacement therapy |
| CRP | [[39](#_ENREF_39)] Worns et al., 2006 | Germany  (Twin study) | 168 twin pairs (108 MZ and 60 same-sex DZ twins) | — | 22% | Age, BMI, smoking, and alcohol consumption |
| CRP | [[40](#_ENREF_40)] Wessel et al., 2007 | USA  (Twin study) | 229 twin pairs | — | 56 % | Age and sex |
| CRP | [[41](#_ENREF_41)] Su et al., 2008 | Vietnam  (Twin study) | 166 male twin pairs (88 MZ twins and 78 DZ twins) | — | 61% | — |
| CRP | [[42](#_ENREF_42)] Sas et al., 2014 | Netherlands  (Twin study) | 214 Dutch female twins (125 MZ twins and 89 DZ twins | Intra-correlation coefficient ICC (95% CI)  MZ pairs, 0.61 (0.40, 0.75)  DZ pairs, 0.36 (0.08, 0.51) | 52% | — |
| Fibrinogen | [[43](#_ENREF_43)] Hamsten et al., 1987 | Sweden  (Family study) | 170 families (85 patient families and 85 control families) | — | 51% | — |
| Fibrinogen | [[44](#_ENREF_44)] Friedlander et al., 1995 | Israel  (Family study) | 465 health subjects (45-64 yrs) | Intra-familial correlation coefficient   \| Type \| Pairs \| Coefficient \| \| --- \| --- \| --- \| \| Spouse \| 123 \| 0.16 \| \| Pa-off \| 341 \| 0.24 \| \| Sibling \| 126 \| 0.28 \| | — | — |
| Fibrinogen | [[45](#_ENREF_45)] Pankow et al., 1998 | USA  (Family study) | 512 randomly ascertained families comprising 2,029 adults | Intra-familial correlation coefficient   \| Type \| Pairs \| Coefficient ±SE \| \| --- \| --- \| --- \| \| Spouse \| 233 \| 0.08±0.06 \| \| Pa-off \| 952 \| 0.15±0.02 \| \| Sibling \| 1619 \| 0.15±0.03 \| | 34% | Age and sex |
| Fibrinogen | [[46](#_ENREF_46)] Souto et al., 2000 | Span  (Family study) | 21 Spanish families comprising 397 individuals | — | 33.6% | — |
| Fibrinogen | [[47](#_ENREF_47)] Freeman et al., 2002 | North European  (Family study) | 89 white European families comprising 537 individuals | — | 28% | Age, sex, SNP, BMI, lifestyle, and metabolic variables |
| Fibrinogen | [[48](#_ENREF_48)] Yang et al., 2003 | USA  (Family study) | 330 families comprising 1,193 individuals | — | 24% | Age [including squared and cubic terms], BMI, cardiovascular disease, diabetes, hypertension treatment, diastolic and systolic blood pressure, alcohol consumption, cigarette smoking, estrogen usage, and genotype of HindIII β-148 polymorphism |
| Fibrinogen | [[24](#_ENREF_24)] Best et al., 2005 | American Indians  (Family study) | 1,294 American Indian relative pairs comprising 1,393 individuals | — | 34% | Age, sex, cohort center, history of cardiovascular disease, physical activity, percent Indian heritage, BMI, waist-hip ratio, blood pressure, hypertension status, smoking, diabetes status, triglyceride, LDL-C, HDL-C |
| Fibrinogen | [[28](#_ENREF_28)] Saunders CL 2006 | England  (Family study) | 9,237 families from a national health survey, comprising 22,297 individuals | \| Type \| Pairs \| Coefficient ±SE \| \| --- \| --- \| --- \| \| Spouse \| 1,102 \| 0.20±0.03 \| \| Pa-off \| 762 \| 0.27±0.04 \| \| Sibling \| 107 \| 0.18±0.09 \| | 23% | — |
| Fibrinogen | [[26](#_ENREF_26)] Friedlander et al., 2006 | Israel  (Family study) | 142 kindreds comprising 881 individuals | — | 42%  (polygenic effect) | Age and sex |
| Fibrinogen | [[49](#_ENREF_49)] Nowak-Gottl et al., 2008 | Germany  (Family study) | 282 white pediatric stroke families comprising 1,002 individuals | — | 22.5% | Age, sex, blood group, smoking, and hormonal contraceptives |
| Fibrinogen | [[33](#_ENREF_33)] Neijts et al., 2013 | Netherland  (Family study) | 3,534 twins, 1,568 of their non-twin siblings, and 2,227 parents from 3,095 families | — | 46% | Age, sex, BMI, smoking status, current and recent illness, anti-inflammatory medication, female sex hormone status, month of data collection, and batch processing |
| Fibrinogen | [[34](#_ENREF_34)] Reding-Bernal et al., 2017 | Mexico  (Family study) | 32 extended families comprising 585 individuals | — | 64% | Age, sex and BMI |
| Fibrinogen | [[50](#_ENREF_50)] Williams PT 2022 | USA  (Family study) | 5,689 offspring-parent pairs and 1,932 sibships in non-Hispanic white families | — | 47.5% | Age and sex |
| Fibrinogen | [[51](#_ENREF_51)] de Lange et al., 2001 | England  (Twin study) | 1,002 female Caucasian twins (149 MZ pairs, 352 DZ pairs) | Intra-correlation coefficient ICC (95% CI)  MZ pairs = 0.51; DZ pairs = 0.34 | 44% | — |
| Fibrinogen | [[35](#_ENREF_35)] Retterstol et al., 2003 | Norway  (Twin study) | 155 MZ twins (68 male pairs and 87 female pairs) | Intra-correlation coefficient: 0.27 | — | — |
| Fibrinogen | [[36](#_ENREF_36)] de Maat et al., 2004 | Denmark  (Twin study) | 282 Danish twins (129 MZ pairs, 153 DZ same-sex twins pairs) | Intra-correlation coefficient ICC (95% CI)  MZ pairs, 0.30 (0.19, 0.41)  DZ pairs, 0.19 (0.16, 0.28) | 21% | Age, sex and BMI |
| Fibrinogen | [[52](#_ENREF_52)] Bladbjerg et al., 2006 | Denmark  (Twin study) | 285 Danish twins (130 MZ twins and 155 DZ twins) | Intra-correlation coefficient ICC (95% CI)  MZ pairs, 0.32 (0.15, 0.47)  DZ pairs, 0.20 (0.04, 0.35) | 34% | Age, gender, BMI, and smoking habits |
| Fibrinogen | [[41](#_ENREF_41)] Su et al., 2008 | Vietnam  (Twin study) | 166 male twin pairs (88 MZ twins and 78 DZ twins) | — | 52% | — |
| Fibrinogen | [[42](#_ENREF_42)] Sas et al., 2014 | Netherlands  (Twin study) | 214 Dutch female twins (125 MZ twins and 89 DZ twins) | Intra-correlation coefficient ICC (95% CI)  MZ pairs, 0.65 (0.48, 0.77)  DZ pairs, 0.19 (-0.07, 0.42) | 67% | — |
| cIMT | [[53](#_ENREF_53)] Duggirala et al., 1996 | Mexico  (Family study) | 46 sibships comprising 88 individuals for CCA; 44 sibships comprising 72 individuals for ICA | — | CCA: 91.6%  ICA: 85.6% | Age, age^2^, age × sex, and age^2^ × sex |
| cIMT | [[54](#_ENREF_54)] Zannad et al., 2001 | France  (Family study) | 89 families comprising 369 subjects | — | 30% | — |
| cIMT | [[55](#_ENREF_55)] Xiang et al., 2002 | Latino Americans  (Family study) | 54 Latino families (hypertensive parent with ≥2 adult offspring) | — | 34% | Age, sex, systolic blood pressure, and cholesterol |
| cIMT | [[56](#_ENREF_56)] Lange et al., 2002 | USA  (Family study) | 122 families comprising 252 individuals with type 2 diabetes | — | 40.8% | Age, sex, race, total cholesterol, hypertension status, and smoking status |
| cIMT | [[57](#_ENREF_57)] North et al., 2002 | American Indians  (Family study) | 32 extended American Indian families comprising 887 individuals | — | 21% | Age, sex, smoking, and diabetes |
| cIMT | [[58](#_ENREF_58)] Fox et al., 2003 | USA  (Family study) | 586 extended families with 1630 sib pairs | Sibling Correlation Coefficients  CCA: 0.17  ICA: 0.14 | CCA: 39%  ICA: 31% | Age, blood pressure, smoking, lipid profiles, BMI, diabetes, antihypertensive treatment, and menopausal status and hormone replacement therapy in women |
| cIMT | [[59](#_ENREF_59)] Juo et al., 2004 | Caribbean Hispanic Americans  (Family study) | 77 Caribbean Hispanic families comprising 440 individuals | — | 40% | Age and sex |
| cIMT | [[38](#_ENREF_38)] Moskau et al., 2005 | Germany  (Family study) | 154 families comprising 565 individuals (106 spouse, 305 offspring) | — | 61% | Age, sex, arterial hypertension, diabetes, and lipoprotein(a) |
| cIMT | [[60](#_ENREF_60)] Wang et al., 2005 | Mexican Americans  (Family study) | 91 Mexican American families with 274 adult offspring | — | 40% | Age, age^2^, sex, systolic blood pressure, fasting insulin, and smoking. |
| cIMT | [[61](#_ENREF_61)] Mayosi et al., 2005 | England  (Family study) | 224 white British families comprising 854 individuals | — | 24% | Age, sex, BMI, and physical exercise |
| cIMT | [[62](#_ENREF_62)] Sayed-Tabatabaei et al., 2005 | Netherlands  (Family study) | 20 extended families comprising 930 individuals | — | 34.8% | Age, sex, BMI, systolic and diastolic blood pressures, LDL, HDL, fasting glucose, smoking, and heart rate |
| cIMT | [[63](#_ENREF_63)] Kao et al., 2005 | Mexican Americans  (Family study) | 24 Mexican American families comprising 620 nondiabetic individuals | — | 16% | Age and sex |
| cIMT | [[64](#_ENREF_64)] Ryabikov et al., 2007 | Russian  (Family study) | 81 Caucasian ancestry nuclear families comprising 286 individuals | \| Type \| Pairs \| Coefficient (95% CI) \| \| --- \| --- \| --- \| \| Spouse \| 48 \| -0.01 (-0.381, 0.365) \| \| Pa-off \| 246 \| 0.10 (-0.035, 0.225) \| \| Sibling \| 81 \| 027 (0.010, 0.492) \| | 54% | Age, sex, BMI, systolic blood pressure, cholesterol, HDL, hypertension medication, diabetes, lifestyle factors |
| cIMT | [[65](#_ENREF_65)] Chien et al., 2008 | Chinese  Taiwan  (Family study) | 62 high LDL families comprising 360 subjects | \| Type \| Pairs \| Coefficient \| \| --- \| --- \| --- \| \| Spouse \| 45 \| 0.389 \| \| Pa-off \| 266 \| 0.376 \| \| Sibling \| 236 \| 0.354 \| | 18.5% | — |
| cIMT | [[66](#_ENREF_66)] Chen et al., 2008 | Hispanic Americans  (Family study) | 149 hypertensive Hispanic American families comprising 603 individuals (179 sibships, 495 offspring) | — | 37% | Age, sex, and BMI |
| cIMT | [[67](#_ENREF_67)] Rampersaud et al., 2008 | Amish Americans  (Family study) | 88 larger Amish pedigrees comprising 478 individuals | — | 29% | Age, age^2^, age × sex, and age^2^ × sex |
| cIMT | [[68](#_ENREF_68)] Sacco et al., 2009 | Dominican Republic  (Family study) | 110 families (Dominican Republic 100, Puerto Rico 4, Cuba 2, Ecuador 2, Nicaragua 1, and Colombia 1) comprising 252 individuals | — | 62% | Age, age^2^, sex, cigarette pack-year, waist hip ratio, and BMI |
| cIMT | [[69](#_ENREF_69)] Li et al., 2013 | Afro-Caribbeans  (Family study) | 7 large Afro-Caribbean families from Tobago comprising 402 individuals | — | 47% | Age and sex |
| cIMT | [[70](#_ENREF_70)] Kuipers et al., 2013 | Afro-Caribbeans  (Family study) | 7 large Afro-Caribbean families from Tobago comprising 395 individuals | — | 35% | — |
| cIMT | [[71](#_ENREF_71)] Ryder et al., 2017 | USA  (Family study) | 558 parents and 369 of their offspring | — | 29% | Age, sex, race, BMI, smoking, and mean arterial pressure |
| cIMT | [[72](#_ENREF_72)] Ware et al., 2022 | Africa  (Family study) | 65 families comprising 130 adults and 65 children | — | 23% | Age, sex, and BMI |
| cIMT | [[73](#_ENREF_73)] Diego et al., 2023 | Mexican Americans  (Family study) | 42 extended Mexican American families comprising 1,446 individuals | — | 28% | Age and sex |
| cIMT | [[74](#_ENREF_74)] Swan et al., 2003 | Scotland  (Twin study) | 264 twin subjects (142 MZ and 122 DZ) | — | Non-significant | — |
| cIMT | [[75](#_ENREF_75)] Zhao et al., 2007 | USA  (Twin study) | 98 male twins (58 MZ and 40 DZ) | Intraclass correlation for carotid IMT  MZ twin pairs: 0.66; (95% CI, 0.62 – 0.69)  DZ twin pairs: 0.37; (95% CI, 0.29 – 0.44) | 59% | Age, systolic blood pressure, and HDL |
| cIMT | [[76](#_ENREF_76)] Lee et al., 2012 | Korea  (Twin study) | 294 twin pairs (236 MZ and 58 DZ) | — | CCA: 48%  ICA: 45%  Bif: 38% | Age and sex |
| cIMT | [[77](#_ENREF_77)] Medda et al., 2014 | Italy  (Twin study) | 348 Italian twin pairs (82 MZ pairs, 92 DZ pairs) | — | 32% | ACE model |
| cIMT | [[78](#_ENREF_78)] Cecelja et al., 2018 | England  (Twin study) | 762 female twins | — | 49% | Age |

Pa-off: parent-offspring; MZ: monozygotic twin pairs; DZ: dizygotic twin pairs; CCA: common carotid artery; Bif: bifurcation artery; ICA: internal carotid artery;

BMI: body mass index; HDL: high-density lipoprotein cholesterol; LDL: Low-density lipoprotein cholesterol.
